# Clinical value of CT-based patient-specific 3D preoperative design combined with conventional instruments in primary total knee arthroplasty: a propensity score-matched analysis

**DOI:** 10.1186/s13018-020-02123-5

**Published:** 2020-12-09

**Authors:** Kai Lei, Li Ming Liu, Yi Xiang, Xin Chen, Hua Quan Fan, Yang Peng, Jiang Ming Luo, Lin Guo

**Affiliations:** 1 Center for Joint Surgery, Southwest Hospital, Third Military Medical University, No. 30 Gaotanyan Street, Shapingba District, Chongqing, 400036 China; 2 Department of Orthopaedics, Logistic Support Forces of the Chinese PLA 985 Hospital, No. 30 Qiaodong Street, Taiyuan, 030001 Shanxi China

**Keywords:** Total knee arthroplasty, Propensity score matching, Three-dimensional, Alignment, VAS, KSS

## Abstract

**Purpose:**

To evaluate the operation and early clinical effect in primary total knee arthroplasty (TKA) about the novel combination of CT-based patient-specific three-dimensional (3D) preoperative design and conventional osteotomy instruments, compared with the conventional method.

**Methods:**

After a 1:1 propensity score-matching (PSM), patients were matched to the novel technique group and the conventional group, 109 cases in each group. The conventional group adopted a preoperative design based on a full-length radiograph (FLX) and received TKA with conventional osteotomy instruments. The novel technique group used a CT-based patient-specific 3D preoperative design combined with conventional osteotomy instruments; during the surgery, the femoral entry point, femoral valgus osteotomy angle, the fix point of tibial plateau extramedullary guide pin, and the position of tibial extramedullary positioning rod were accurately selected according to the preoperative 3D design to ensure accurate intraoperative implementation. The lower limb alignment, component position, operation time, tourniquet time, hospital stay, blood loss volume, incidence of postoperative complications, visual analog scale (Vas) score, and New Knee Society Score System (NEW-KSS) at 1 day before operation and 1, 6, and 12 months after operation were recorded and compared.

**Results:**

The novel technique group was significantly better than the conventional group in controlling lateral tibial component angle (LTC) (*P* < 0.001), and the novel technique group had lower percentages of hip-knee-ankle angle (HKA) outliers (*P* < 0.001) and overcorrection (*P* = 0.003). The operation time, tourniquet time, and hospital stay of the novel technique group was shorter (*P* < 0.05). In 1 month after the operation, the novel technique group achieved a significantly better VAS score (*P* < 0.05), but a similar NEW-KSS score (*P* > 0.05) when compared with the conventional group. But in 6 and 12 months after surgery, no statistical differences were seen in the above two scores (*P* > 0.05).

**Conclusion:**

The novel technique of CT-based patient-specific 3D preoperative design combined with conventional instruments can improve the accuracy of osteotomy in primary total knee arthroplasty, with benefits of significantly reducing pain and rapid recovery during the early postoperative period, but having no obvious effect on outcome after a 1-year follow-up.

## Introduction

Restoring the neutral mechanical alignment is usually considered as one of the prerequisites for a successful total knee arthroplasty (TKA). Previous literature has shown that malalignment or malposition of the components are often closely associated with complications such as polyethylene wear and aseptic loosening [1–3]. However, the incidence of the lower limb malalignment or malposition of the components after conventional TKA is as high as 20–30% [4, 5].

To overcome the limitations of the conventional method for TKA, many modified surgical techniques have been adopted. The proponents of computer navigation believe that this method can achieve more accurate osteotomy by adding about 10 min of registration time during the surgery [6, 7]. And robotics can reduce the occurrence of outliers in the lower limb [8–10]. However, compared with conventional methods, there are deficiencies in computer navigation and robotics such as longer surgical time [8, 11], higher surgery costs [8, 12], a substantial learning curve [8, 10], and more complications. Furthermore, during the surgery, the percentage of temporarily changing from robotic technology to conventional method due to various reasons is as high as 22% [13]. Patient-specific instrumentation (PSI) is regarded to enable better component positions and lower limb alignment with decreased operative steps, less blood loss and fat embolism, and shorter operative time [14, 15]. However, more and more literatures have shown that compared with the conventional method, PSI has no obvious advantages in lower limb alignment, component position, and postoperative knee function [14, 16–19]. Some authors even argued that PSI was worse at the control of the lateral tibial component (LTC) angle [17–20].

In this study, on the basis of summarizing the principles, advantages, and disadvantages of the conventional method and the modified techniques, we adopted a novel technique of CT-based patient-specific three-dimensional preoperative design combined with conventional osteotomy instruments. The advantages of this technique include personalized preoperative design, precise intraoperative positioning, no need to purchase new equipment, better control of the surgical time and cost, and easier application due to similar procedures with the conventional method.

Patients were divided into the novel technique group and the conventional group to evaluate the operation and early clinical effect of the novel technique compared with the conventional method.

## Materials and methods

### Patients

Relevant medical records and examination results of patients admitted to our center from January 2016 to June 2019 who met the inclusion criteria and did not meet the exclusion criteria were retrospectively collected. The novel technique group included those who had received novel surgery with patient-specific 3D preoperative design, and the conventional group included those who had received conventional surgery with conventional preoperative design. The exclusion criteria in both the novel technique group and the conventional group were (1) patients without full-length radiographs (FLX) before and after surgery; (2) patients with any diseases that affected the alignment of the lower limbs, such as previous fractures, deformities, and congenital abnormalities; (3) patients with multiple joint lesions or weak bodies that affect the accurate evaluation of function; and (4) patients with incomplete follow-up data. A total of 398 cases were included, of which 196 were in the novel technique group and 202 in the conventional group.

To reduce the effects of selection bias and potential confounding factors [21–24], we used age, gender, left and right, etiology, ethnicity, body mass index (BMI), and preoperative hemoglobin to perform a one-to-one propensity score matching (PSM) using a 0.02-caliper width. In the end, 109 patients were matched in each group by PSM.

### Preoperative design and surgical techniques

The conventional group adopted a preoperative design based on a full-length radiograph (FLX) and used nerve block anesthesia. With the patient in the supine position, a pad was placed under the foot to help maintain knee flexion before disinfection and draping. A midline incision of 12–14 cm was cut, and the Insall’s medial parapatellar approach was conducted crossing the patellar surface to avoid cutting the fibers of the patellar extensor apparatus. Part of the fat pad was removed to complete the exposure. After the dislocation of the tibia, the distal end of the extramedullary positioning rod was aimed with the second toe and the proximal end with 1/3 of the tibial tubercle. The position of the positioning rod and the retroversion angle of the components were evaluated. An osteotomy of 8–10 mm was first conducted referring to the lateral plateau in the varus knees, and an osteotomy of 6–8 mm was first conducted referring to the medical plateau in the valgus knees. For distal femoral osteotomy, the apex of the intercondylar notch was used as the intramedullary positioning rod entry point, and the appropriate valgus angle was selected according to the hip-knee-shaft angle (HKS) measured on the FLX to perform a standard distal osteotomy of 9 mm. Extramedullary test of the lower limb alignment was performed; the cruciate ligament, osteophyte, and residual meniscus tissues were removed to balance the extension gap, and appropriate lateral release was conducted if necessary as described by Kim and Ranawat [25, 26]. After the extension gap was well balanced, the femoral osteotomy at the flexion position was conducted referring to the Whiteside’s line, then referring to the tibial plateau osteotomy surface and the extension gap to balance the flexion gap. After the four-in-one osteotomy, femoral intercondylar osteotomy, and lateral tibial preparation were completed, the test model of the component was installed to determine the lateral stability at the flexion position. After obtaining a stable balance, bone cement was used to fix the component, and the polyethylene pad was installed. The flexion-extension gap was tested after the bone cement was set. Finally, the joint cavity was rinsed, the drainage tube was placed, and the incision was closed. A tourniquet was applied before the skin incision and released after closing the joint capsule. In order to reduce total blood loss, tranexamic acid was routinely used in TKA.

**Fig. 1:**
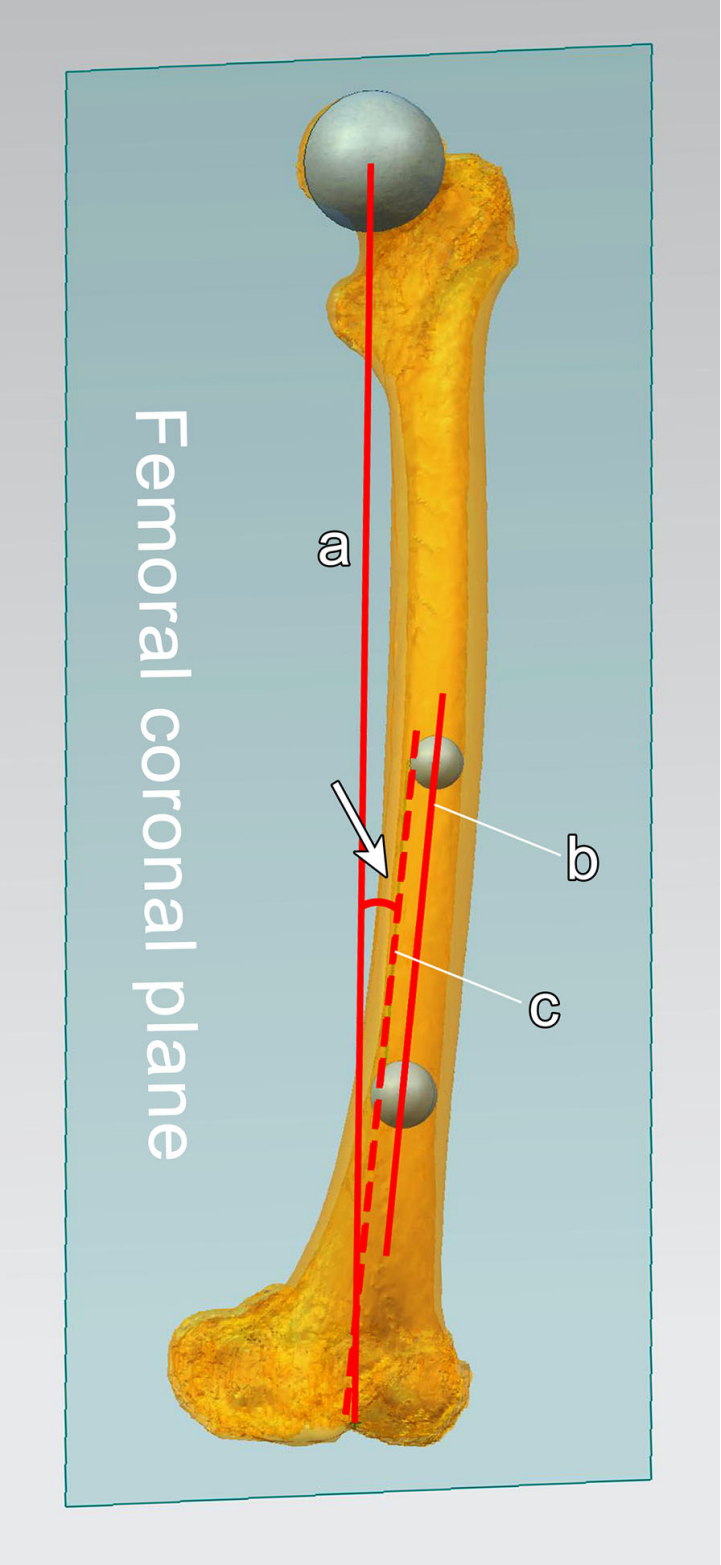
The projection angle of the hip-knee-shaft on the coronal plane. The blue plane in the figure was the femoral coronal plane (the femoral mechanical axis was in this coronal plane, and the surgical trans-epicondylar axis was parallel to the coronal plane); line a was the femoral mechanical axis, line b was the anatomical axis of the distal femur, and line c was the coronal projection of the distal femoral anatomical axis; the angle between line a and line c is the projection angle of the HKS on the coronal plane

**Fig. 2:**
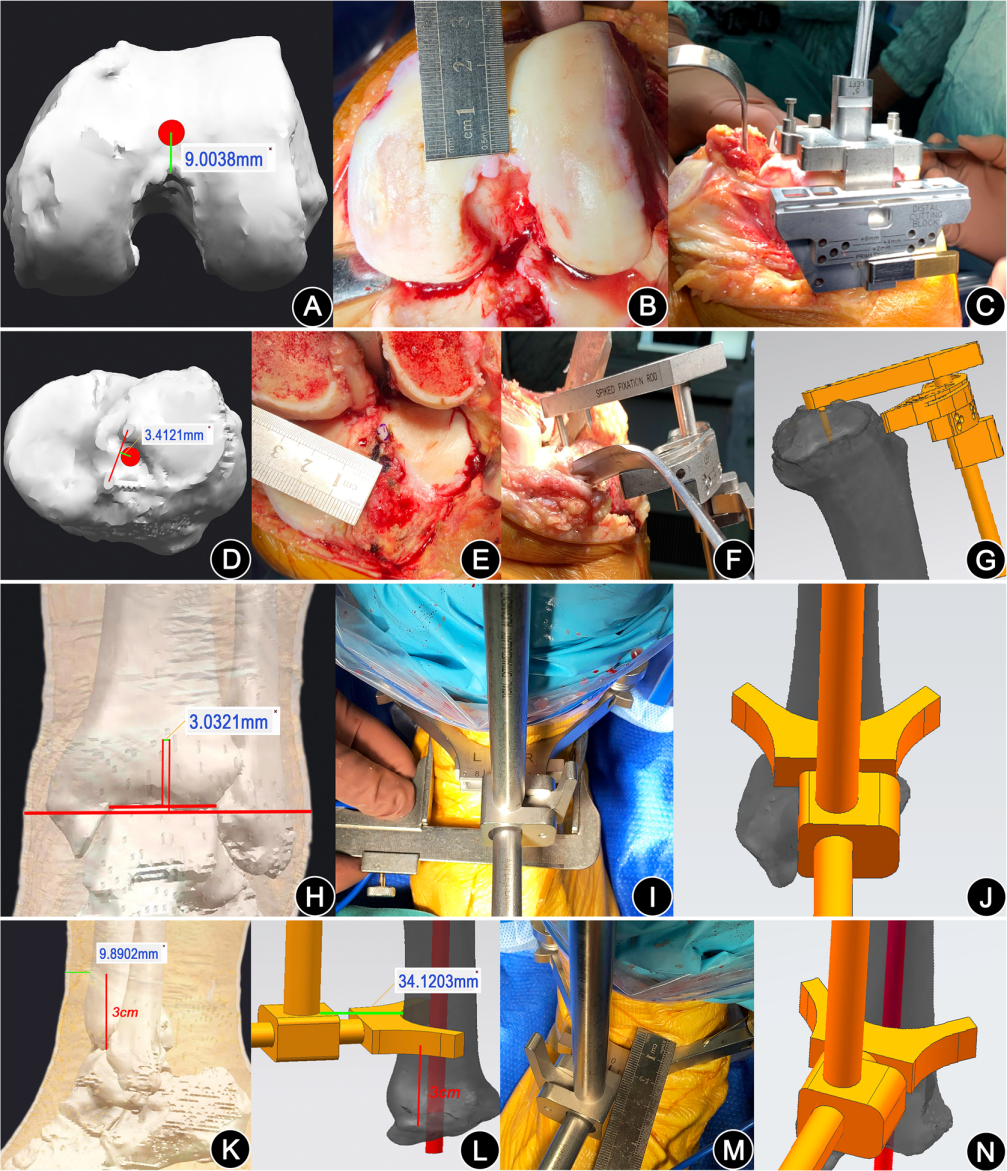
Illustration of the key steps of the novel technology group. **a**–**c** The distal femoral osteotomy. The red dot in **a** was the position where the anatomical axis of the distal femur penetrated the distal femur cortex on the preoperative 3D model, i.e., the femoral entry point; **b** the accurate positioning of the femoral entry point during surgery; **c** the intramedullary positioning rod was inserted from the femoral entry point into the medullary cavity for about 20 cm, and the distal femur osteotomy was performed according to the projection angle of the HKS on the coronal plane. **d**–**g** The positioning of the proximal end of the tibial extramedullary rod. The red dot in **d** was the position where the tibial anatomical axis penetrated the tibial plateau cortex, i.e., the fix point of the tibial plateau extramedullary guide pin; **e** the accurate positioning of the above anchor point during surgery; **f, g** the tibial extramedullary guide pin was fixed to the anchor point to ensure the accurate positioning of the extramedullary positioning rod at the proximal end of the tibia during the surgery and on the preoperative 3D models, respectively. **h**–**j** The coronal positioning of the distal end of the tibial extramedullary rod; **h** distance between the midpoint of the ankle mortise and the midpoint of medial and lateral malleolus; **i, j** the accurate positioning of the distal end of the tibial extramedullary rod on the coronal plane with reference to the above preoperative design during the surgery and on the preoperative 3D model, respectively. **k**–**n** The sagittal positioning of the distal end of the tibial extramedullary rod. **k** the thickness of the anterior tibial soft tissue at 3 cm above the ankle mortise; **l** the distance between the anterior tibial cortex and the distal tibial extramedullary positioning rod; **m, n** the accurate positioning of the distal end of the tibial extramedullary rod on the sagittal plane was ensured with reference to the distance between the anterior tibial skin and the distal tibial extramedullary positioning rod (the distance between the anterior tibial cortex and the distal tibial extramedullary positioning rod minus the thickness of the anterior tibial soft tissue) during the surgery and on the preoperative 3D model, respectively

**Fig. 3:**
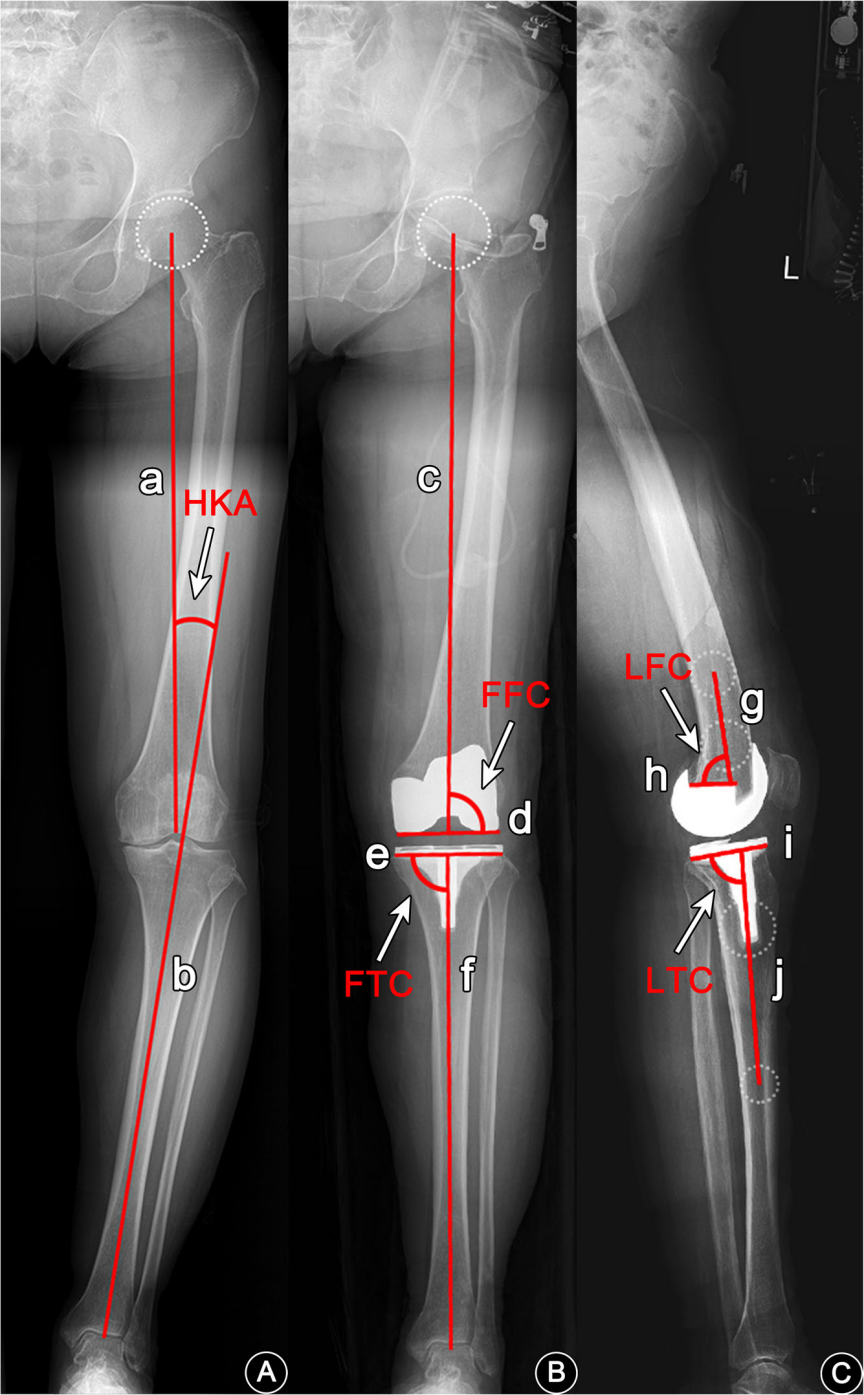
Measurement of HKA, FFC, FTC, LFC, and LTC. HKA hip-knee-ankle angle, FFC frontal femoral component angle, FTC frontal tibial component angle, LFC lateral femoral component angle, LTC lateral tibial component angle. **a** Line a was the femoral mechanical axis, and line b was the tibial mechanical axis; the acute angle formed between them was recorded as the preoperative HKA. **b** Line c was the postoperative femoral mechanical axis, line d was the line across the bottom of the femoral condyles, line e was the line across the bottom of the tibial plateau on the anteroposterior radiograph, and line f was the postoperative tibial mechanical axis; the lateral angle between line c and line d was recorded as frontal femoral component angle (FFC), the medial angle between line e and line f was recorded as frontal tibial component angle (FTC), and the acute angle between line c and line f was recorded as the postoperative HKA. **c** Line g was the line connecting the center points of the femoral shaft at 0 cm and 5 cm above the implant, line h was the line across the bottom of the femoral implant, line i was the line across the bottom of the tibial plateau on the lateral radiograph, and line j was the line connecting the center points of the tibial shaft at 5 cm and 15 cm below the joint line; the posterolateral angle between line g and line h was recorded as lateral femoral component angle (LFC), and the posterolateral angle between line i and line j was recorded as lateral tibial component angle (LTC)

The novel technique group used the full-length computed tomography (CT) data of the patients (thin scan of 1 mm at the knee joint and thick scan of 3 mm at the rest parts) and performed 3D reconstruction with Mimics Research 19.0. With the CATIA 5.20 and NX12.0 software, the engineer and the surgeon at our center formulated the patient-specific 3D preoperative design. The best spherical fitting of the femoral head was performed, and the line between the obtained center point and the apex of the femoral intercondylar notch was defined as the mechanical axis of the femur; the line connecting the center points of the femoral medullary cavity 10 cm and 20 cm above the knee joint line was defined as the anatomical axis of the distal femur [27, 28]; the line between the most prominent point of the lateral femoral epicondyle and the most concave point of the medial femoral epicondyle was the surgical trans-epicondylar axis (sTEA) [29]. The plane defined by the femoral mechanical axis and sTEA was recorded as the femoral coronal plane (the femoral mechanical axis was in this coronal plane, and sTEA was parallel to the coronal plane), the plane perpendicular to the femoral mechanical axis was recorded as the transverse plane, and the line connecting the center points of the tibial medullary cavity at 5 cm below the tibial tubercle and 5 cm above the ankle mortise was defined as the tibial anatomical axis. In the preoperative design, the key information needed to be obtained were (1) the projection angle of the HKS on the coronal plane (Fig. 1); (2) the position where the distal femoral anatomical axis penetrated the distal femur, which was recorded as the femoral entry point (Fig. 2a); (3) the projection angle of the sTEA and the posterior femoral condylar tangents on the transverse plane, which was recorded as the posterior condylar angle (PCA); (4) the position where the tibial anatomical axis penetrated the tibial plateau, which was recorded as the fix point of the tibial plateau extramedullary guide pin (Fig. 2d); (5) distance between the midpoint of the ankle mortise and the midpoint of the medial and lateral malleolus (Fig. 2h); (6) thickness of the anterior tibial soft tissue at 3 cm above the ankle mortise (Fig. 2k) and the distance between the anterior tibial cortex and the distal tibial extramedullary positioning rod (Fig. 2l); and (7) the volume of the femoral and tibial osteotomy. During the surgery, the femoral entry point was selected strictly according to the preoperative plan (Fig. 2a, b); the osteotomy angle was selected based on the projection angle of the HKS on the coronal plane (Fig. 2c), and the distal osteotomy was guided according to the specific osteotomy volume. Based on the preoperative plan, the fix point of the tibial plateau extramedullary guide pin was selected to determine the proximal end of the extramedullary positioning rod (Fig. 2d-g). The thickness of the anterior tibial soft tissue at 3 cm above the ankle mortise (Fig. 2k), the distance between the anterior tibial cortex and the distal tibial extramedullary positioning rod (Fig. 2l) as well as the distance between the midpoint of the ankle mortise and the midpoint of the medial and lateral malleolus (Fig. 2h) were used to determine the distal end of the rod (Fig. 2h-n). The tibial osteotomy was conducted according to the osteotomy volume. The femoral rotatory osteotomy was conducted referring to the PCA. The remaining surgical techniques were the same as the conventional group.

For all patients, the aim was to restore the neutral lower limb alignment on the coronal plane and achieve 90° of the lateral femoral component angle (LFC) and 3° of the lateral tibial component angle (LTC) on the sagittal plane (Fig. 3). The surgeries in both the conventional group and the novel technique were performed by the same senior surgeon at our center, and the same primary posterior-stabilized component was used (LEGION Total Knee System, Smith-Nephew, Inc., Memphis, IN, USA). Except for the preoperative design and surgical technique, the two groups had the same surgical procedures, postoperative pain management, rehabilitation training, and discharge standards.

### Outcome measurements and data collection

The weight-bearing FLX before and after surgery (the preoperative ones were taken within 1 month before surgery and the postoperative ones 3 days after surgery) of the patients were collected via the picture archiving and communication system (PACS) and imported into Autodesk AutoCAD 2019 for measurement of preoperative hip-knee-ankle (HKA) angles and postoperative frontal femoral component angle (FFC), lateral femoral component angle (LFC) [30], frontal tibial component angle (FTC), lateral tibial component angle (LTC) [31, 32], and HKA angles (Fig. 3). Three surgeons specializing in TKA performed blind measurement twice; in order to ensure that the raters had sufficient forgetting time, each was required to measure at an interval for more than 2 weeks [33, 34]. For HKA, the varus was defined as negative and the valgus positive; for LTC, the retroversion was positive and the anteversion negative. Values exceeding the target value by 3° were recorded as outliers, and the percentage of outliers was calculated. Patients with varus knee preoperatively who had HKA valgus greater than 1° after surgery and patients with valgus knee preoperatively who had HKA varus greater than 1° after surgery were recorded as overcorrected, and the percentage of overcorrection was calculated.

Medical records such as the operation time, tourniquet time, hospital stay, intraoperative bleeding, incidence of postoperative complications, hemoglobin value 1 day before the operation, 1 day after the operation, and 3 days after operation were collected via the electronic medical record management system. And visual analog scale (VAS) score and New Knee Society Score (NEW-KSS) [35] before operation and 1, 6, and 12 months after operation were recorded.

This study has been approved by the local Ethics Committee (No. KY2019123).

### Data analysis

The intraclass correlation coefficient (ICC) was used to evaluate the consistency of the inter-rater and the intra-rater measurements; chi-squared test was used to test the differences of the classification data and independent *t* test was applied to check the measurement data between two groups. Repeated measures analysis of variance (ANOVA) was used, to determine any significant differences between VAS and New-KSS scores obtained at set time intervals before and after TKA. The statistical significance of the differences was all set at 0.05, and the statistical software was SPSS 25.0.

## Results

### Preoperative data

Before PSM, the differences of age, gender, etiology, preoperative hemoglobin, and preoperative HKA were statistically significant between the two groups (*P* < 0.05). After PSM, all preoperative baseline data were not significantly different between the two groups (Table 1).

**Table 1:** Patient characteristics before and after propensity score matching (PSM)

Characteristics	Before PSM (*n* = 398)			After PSM (*n* = 218)		
	Conventional group (*n* = 202)	Novel technique group (*n* = 196)	*P*	Conventional group (*n* = 109)	Novel technique group (*n* = 109)	*P*
Gender (male to female)	31:171	52:144	**0.006**^**b**^	20:89	25:84	0.403^b^
Side (left to right)	101:101	95:101	0.760^b^	53:56	52:57	0.892^b^
Ethnicity (Han to other)	198:4	188:8	0.351^**b**^	106:3	105:4	1.000^b^
Etiology (OA to RA to others)	165:31:6	182:11:3	**0.003**^**b**^	104:4:1	97:10:2	0.180^b^
Age (1:2:3:4:5)	16:68:101:14:3	5:11:78:86:16	**< 0.001**^**b**^	1:14:77:14:3	5:11:75:15:3	0.573^b^
BMI (kg/m^2^)	25.43 ± 3.31	25.60 ± 3.23	0.604^a^	25.56 ± 3.34	25.60 ± 3.42	0.937^a^
Hb preoperative (g/L)	127.25 ± 14.53	130.90 ± 14.46	**0.013**^**a**^	130.68 ± 12.36	130.28 ± 14.61	0.830^a^
HKA preoperative (degree)	− 6.86 ± 10.60	− 9.39 ± 8.79	**0.010**^**a**^	− 9.17 ± 10.05	− 9.30 ± 8.60	0.916^a^

Age is divided into 5 levels, level 1: < 50 years, level 2: 50–59 years, level 3: 60–69 years, level 4: 70–79 years, level 5: > 79 years

^**a**^Stands for *t* test

^**b**^Stands for chi-squared test

### Surgical data

No serious complications such as infection or loosening of the components occurred during the follow-up period. The operation time of the novel technique group was significantly shorter than that of the conventional group [(62.14 ± 9.94) min vs (75.52 ± 18.59) min, *P* < 0.001]. The tourniquet time of the novel technique group was significantly shorter than that of the conventional group [(36.29 ± 7.29) min vs (49.29 ± 13.46) min, *P* < 0.001]. The hospital stay in the novel technique group was shorter than that in the conventional group, and the difference was statistically significant [(8.40 ± 1.69) day vs (8.79 ± 1.57) day, *P* = 0.017]. There was no statistical difference in the intraoperative bleeding and the degree of postoperative hemoglobin change between the two groups (Table 2).

**Table 2:** Comparison of surgical data

	Conventional group (*n* = 109)	Novel technique group (*n* = 109)	*P*
Operation time (min)	75.52 ± 18.59	62.14 ± 9.94	**< 0.001**
Tourniquet time (min)	49.29 ± 13.46	36.29 ± 7.29	**< 0.001**
Length of hospital stay (day)	8.72 ± 1.12	8.40 ± 1.69	**0.017**
Mean intraoperative bleeding (mL)	201.10 ± 42.43	190.73 ± 52.08	0.109
Mean Hb decrease day 1 (g/L)	17.59 ± 8.67	19.50 ± 9.72	0.126
Mean Hb decrease day 3 (g/L)	33.61 ± 11.41	35.46 ± 13.88	0.285

The *t* test was used for all

### Postoperative component position and lower limb alignment

For the measurement of HKA, FFC, LFC, FTC, and LTC (Fig. 3), the inter-rater and intra-rater results showed excellent consistency (ICC > 0.9, *P* < 0.05).

The difference in LTC and the percentage of outliers between the two groups were statistically significant; the LTC control of the novel technique group was better than the conventional group [(4.23 ± 2.57)° vs (9.48 ± 3.74)°, *P* < 0.001), and the percentage of outliers in the novel technique group was significantly smaller than the conventional group (31.68% vs 95.05%, *P* < 0.001). The FTC, FFC, LFC, and percentage of their outliers were similar between the two groups.

The novel technique group had fewer HKA outliers (17.43% vs 40.37%, *P* < 0.001) and overcorrected cases (11.01% vs 26.61%, *P* = 0.003); there was no statistical difference in postoperative HKA between the two groups (Table 3).

**Table 3:** Comparison of postoperative component position and lower limb alignment

	Conventional group (*n* = 109)	Novel technique group (*n* = 109)	*P*
LTC mean ± SD	9.48° ± 3.74°	4.23° ± 2.57°	**< 0.001**^**b**^
LTC percentage of outliers > 3°	95.05%	31.68%	**< 0.001**^**a**^
FTC mean ± SD	89.77° ± 2.11°	89.58° ± 2.00°	0.496^a^
FTC percentage of outliers > 3°	13.86%	14.85%	0.842^a^
LFC mean ± SD	88.31° ± 3.01°	88.51° ± 2.81°	0.615^b^
LFC percentage of outliers > 3°	33.66%	32.67%	0.883^a^
FFC mean ± SD	90.01° ± 2.46°	90.05° ± 1.95°	0.903^b^
FFC percentage of outliers > 3°	18.81%	14.85%	0.455^a^
HKA mean ± SD	− 0.16° ± 3.80°	− 0.46° ± 2.63°	0.557^b^
HKA percentage of outliers > 3°	40.37%	17.43%	**< 0.001**^**a**^
HKA percentage of overcorrection	26.61%	11.01%	**0.003**^**a**^

The target value of LTC is 3°, the target values of FFC, FTC, and LFC are 90°, and the target value of HKA is 0°

*LTC* lateral tibial component angle, *FTC* frontal tibial component angle, *LFC* lateral femoral component angle, *FFC* frontal femoral component angle, *HKA* hip-knee-ankle angle.

^**a**^Stands for chi-squared test

^**b**^Stands for *t* test

### Postoperative functional outcomes

Both the novel technique and the conventional groups demonstrated significant improvements in the functional outcome scores at 12 months (*P* < 0.001) (Table 4).

**Table 4:** Functional outcomes at 1, 6, and 12 months for the two groups

Functional outcomes	Pre-op	1 month	6 months	12 months	*P*
*Conventional group (n = 109)*					
VAS score*	6.34 ± 1.90	4.32 ± 0.65	1.99 ± 0.32	1.03 ± 0.16	< 0.001
KSS objective knee indicators	42.52 ± 12.67	59.07 ± 7.11	63.61 ± 3.25	68.56 ± 12.62	< 0.001
KSS symptom	9.35 ± 3.47	11.47 ± 2.41	19.55 ± 3.31	21.95 ± 1.18	< 0.001
KSS patient satisfaction	18.75 ± 6.45	27.82 ± 7.07	31.48 ± 5.49	37.45 ± 5.17	< 0.001
KSS patient expectations	10.70 ± 1.08	9.31 ± 1.84	11.07 ± 2.42	13.30 ± 2.73	< 0.001
KSS functional activities	34.92 ± 13.85	45.13 ± 12.26	62.96 ± 11.05	79.94 ± 13.96	< 0.001
*Novel technique group (n = 109)*					
VAS score*	6.64 ± 1.71	3.58 ± 0.71	1.98 ± 0.19	1.02 ± 0.13	< 0.001
KSS objective knee indicators	42.28 ± 13.45	59.39 ± 7.37	63.91 ± 2.99	68.38 ± 12.32	< 0.001
KSS symptom	9.05 ± 3.69	11.58 ± 2.33	19.64 ± 2.74	21.85 ± 1.10	< 0.001
KSS patient satisfaction	18.90 ± 5.55	28.39 ± 6.97	31.67 ± 5.43	37.85 ± 3.71	< 0.001
KSS patient expectations	10.65 ± 1.50	9.49 ± 1.78	10.85 ± 2.53	13.67 ± 1.86	< 0.001
KSS functional activities	35.36 ± 11.83	45.51 ± 12.65	63.52 ± 10.65	81.67 ± 5.58	< 0.001

*VAS Lower scores indicate better outcomes. Repeated measures analysis of variance (ANOVA) was used for all

In 1 month after the operation, the novel technique group achieved a significantly better VAS score (3.58 ± 0.71 vs 4.32 ± 0.65, *P* < 0.001), but a similar NEW-KSS score (*P* > 0.05) when compared with the conventional group. And in 6 and 12 months after surgery, no statistical differences were seen in the above two scores (*P* > 0.05) (Table 5).

**Table 5:** Comparison of functional outcomes

	Conventional group (*n* = 109)	Novel technique group (*n* = 109)	*P*
VAS score*			
Pre-op	6.34 ± 1.90	6.64 ± 1.71	0.218
1 month	4.32 ± 0.65	3.58 ± 0.71	**< 0.001**
6 months	1.99 ± 0.32	1.98 ± 0.19	0.797
12 months	1.03 ± 0.16	1.02 ± 0.13	0.653
*KSS o*bjective knee indicators			
Pre-op	42.52 ± 12.67	42.28 ± 13.45	0.889
1 month	59.07 ± 7.11	59.39 ± 7.37	0.751
6 months	63.61 ± 3.25	63.91 ± 2.99	0.475
12 months	68.56 ± 12.62	68.38 ± 12.32	0.914
*KSS* symptom			
Pre-op	9.35 ± 3.47	9.05 ± 3.69	0.533
1 month	11.47 ± 2.41	11.58 ± 2.33	0.732
6 months	19.55 ± 3.31	19.64 ± 2.74	0.824
12 months	21.95 ± 1.18	21.85 ± 1.10	0.515
*KSS* patient satisfaction			
Pre-op	18.75 ± 6.45	18.90 ± 5.55	0.857
1 month	27.82 ± 7.07	28.39 ± 6.97	0.550
6 months	31.48 ± 5.49	31.67 ± 5.43	0.804
12 months	37.45 ± 5.17	37.85 ± 3.71	0.508
*KSS* patient expectations			
Pre-op	10.70 ± 1.08	10.65 ± 1.50	0.795
1 month	9.31 ± 1.84	9.49 ± 1.78	0.478
6 months	11.07 ± 2.42	10.85 ± 2.53	0.512
12 months	13.30 ± 2.73	13.67 ± 1.86	0.248
*KSS* functional activities			
Pre-op	34.92 ± 13.85	35.36 ± 11.83	0.801
1 month	45.13 ± 12.26	45.51 ± 12.65	0.820
6 months	62.96 ± 11.05	63.52 ± 10.65	0.704
12 months	79.94 ± 13.96	81.67 ± 5.58	0.230

*VAS Lower scores indicate better outcomes. *t* test was used for all

## Discussion

The postoperative outcomes of total knee replacement are related to many factors, and the surgical factors often include the placement position of components, the alignment and soft tissue balance of the lower limbs, and the bone cement technique [36]. Among them, the neutral alignment of the lower limbs and the component position may be the key to avoiding the early failures of TKA. In order to obtain the desired postoperative lower limb alignment and components position, technologies such as patient-specific instrumentation, computer navigation, and robot-assisted TKA came into being, but these high-tech technologies are also accompanied by various disadvantages, such as longer operation time [8], higher operation cost [8, 37], more potential complications [13, 38], and the possibility to switch to conventional TKA for various reasons during the operation [11, 13]. In addition, TKA is no longer limited to large hospitals, and many small hospitals have also carried out such operations, but most of the smaller hospitals do not have the equipment or technology related to the above high-tech technologies. Is there a TKA surgical technique that is simpler and more economical than the abovementioned high-tech techniques, but more precise than conventional methods in controlling the alignment of the lower limbs and the position of the components? The novel technique of CT-based patient-specific 3D preoperative design combined with conventional osteotomy instruments provides one possibility.

The LTC of the novel technique group was better than the conventional group [4.23° ± 2.57° vs (9.48° ± 3.74°, *P* < 0.001), and the percentage of outliers was also significantly smaller than the conventional group (31.68% vs 95.05%, *P* < 0.001). The results demonstrated that the novel technology group is superior to the conventional group in controlling the position of the tibial components. The conventional method usually determines the position of the positioning rod and the retroversion angle of the osteotomy instrument by the surgeon’s visual assessment, whereas the novel technique uses the positioning pin of the extramedullary positioning rod to accurately restore the pre-designed fix point of the guide pin and accurately control the position of the distal end of the positioning rod based on the thickness of the soft tissue of the anterior tibial of the patient to ensure the accurate intraoperative implementation of the preoperative design. The precise patient-specific preoperative design and its accurate implementation are the main reasons why the novel technique group has significantly better control of the LTC than the conventional group (*P* < 0.001). The LTC angle after TKA would significantly affect the knee joint movement, the fixation of implants, and the wear of polyethylene pads, etc .[39–41]. Therefore, this angle is one of the important evaluation indexes of the radiographic evaluation of the American Knee Society [31]. Yan et al. randomly divided 90 OA knee joints into three groups and performed a randomized controlled trial on the lower extremity alignment of PSI, computer navigation, and conventional method. They found that the average LTC values and outliers of the three methods had no statistical differences postoperatively [16]. Rhee et al. summarized and evaluated 5 studies on the sagittal alignments of the tibial component and found that the number of outliers in LTC included 131 of 1020 cases in the computer-navigated TKA and 154 of 1026 cases in the conventional TKA group; the difference was not significant (*P* = 0.17) [42]. More researches have indicated that PSI is even worse than the conventional method in controlling LTC [17–20]. In the control of FTC, both groups performed well without statistical differences in this study. Previous studies of computer navigation, PSI, and robotics have shown that in the control of FTC, these more expensive techniques might not have obvious advantages compared with the conventional method [6, 7, 14, 16, 43–45]. This may be because the experienced surgeons in those studies could control FTC well during the surgery without deviation.

The conventional method usually chooses the apex of the intercondylar notch as the medullary entry point and uses the HKS angle measured on FLX for distal femoral osteotomy. Preoperative 3D design can more accurately evaluate the medullary entry point and measure the valgus osteotomy angle. In most patients, the femoral medullary cavity is larger than the intramedullary positioning rod, and the swing of the drill when drilling may cause the intramedullary diameter larger than the diameter of the intramedullary positioning rod, resulting in a 1° deviation [46]; in addition, during distal femoral osteotomy, the osteotomy instrument can only select an integer, which may result in intraoperative selection bias. The above factors have reduced the accuracy of intraoperative implementation of HKS to a certain extent, which may be one of the main reasons for the two groups to have no significant difference in the femoral component position. However, from postoperative results, the control of the femoral component position in the novel technique group and conventional group are both acceptable. Computer-navigated and robotics use technologies such as intraoperative navigation and second calibration to achieve more accurate intramedullary positioning and femoral osteotomy. However, many studies have proven that they are not superior to the conventional method in the femoral component position [16, 42–44, 47]; PSI also does not provide better results in this field [14, 45].

Compared with the conventional group, the novel technique group had a lower percentage of HKA outliers (17.43% vs 40.37%, *P* < 0.001) and percentage of HKA overcorrection (11.01% vs 26.61%, *P* = 0.003), although the mean value of HKA between the two groups was not statistically significant. This demonstrates that the novel technology can reduce the occurrence of lower limb alignment outliers than the conventional one.

The operation time and tourniquet time of the novel technology group were significantly less than those of the conventional group. After excluding the influence of related confounding factors, we believed that the longer tourniquet time in the conventional group was mainly due to repeated adjustment and confirmation of the femoral medullary entry point and tibial positioning system during the surgery, while the novel technology group mainly needed to confirm the positioning system according to the preoperative plan, reducing the incidence of repeated adjustments, thereby reducing both tourniquet time and operation time by about 13 min on average.

There was no significant difference between the two groups in the scores of VAS and NEW-KSS at 6 and 12 months after the operation. However, in the VAS pain score at 1 month after the operation, the novel technology group was better than the conventional group(3.58 ± 0.71 vs 4.32 ± 0.65), and the difference was statistically significant (*P* < 0.05). In each item of the NEW-KSS score 1 month after the operation, the novel technology group had a tendency to be superior to the conventional group, but the difference was not statistically significant (*P* > 0.05). In the case that the two groups of early rehabilitation and pain management were consistent, this indicated that the novel technique group was superior to the conventional group in early pain and recovery, which may be related to the shorter tourniquet time and operation time in the novel technique group.

This study also has some deficiencies. First, it does not include computer-navigated, robotics, or PSI technology to compare with the novel technique. Second, it has not conducted mid- or long-term follow-up and functional scoring of the patients. Third, it is a single-center study and the CT-based patient-specific three-dimensional preoperative design all came from one manufacturer. Multi-center studies and execution with other manufacturers are needed for further verification.

## Conclusion

This novel technique that combines patient-specific 3D preoperative design with conventional osteotomy instruments can better control the position of tibial components, reduce the occurrence of the lower limb alignment outliers, and improve the accuracy of osteotomy in TKA to a certain extent. Meanwhile, the operation time is shorter, and the short-term functional outcomes of patients are satisfactory. However, the functional outcomes for 6 months and 1 year were not significantly different from that of the conventional group. Further follow-up study is needed for the mid- and long-term functional outcomes.

## Data Availability

All data and materials of the present study were in full compliance with the journal’s policy.

## Abbreviations

CT: Computed tomography
3D: Three-dimensional
TKA: Total knee arthroplasty
PSM: Propensity score matching
FLX: Full-length radiograph
VAS: Visual analog scale
NEW-KSS: New Knee Society Score System
PSI: Patient-specific instrumentation
HKS: Hip-knee-shaft angle
HKA: Hip-knee-ankle angle
PCA: Posterior condylar angle
LTC: Lateral tibial component angle
LFC: Lateral femoral component angle
FTC: Frontal tibial component angle
FFC: Frontal femoral component angle
sTEA: Surgical trans-epicondylar axis
ICC: Intraclass correlation coefficient
BMI: Body mass index
PACS: Picture archiving and communication system
